# Review of Animal Models to Study Urinary Bladder Function

**DOI:** 10.3390/biology10121316

**Published:** 2021-12-11

**Authors:** Jing-Dung Shen, Szu-Ju Chen, Huey-Yi Chen, Kun-Yuan Chiu, Yung-Hsiang Chen, Wen-Chi Chen

**Affiliations:** 1 Division of Urology, Department of Surgery, Taichung Armed Forces General Hospital, Taichung 41168, Taiwan; jing-dung@803.org.tw; 2 National Defense Medical Center, Taipei 11490, Taiwan; 3 Graduate Institute of Integrated Medicine, College of Chinese Medicine, China Medical University, Taichung 40402, Taiwan; d888208@ms45.hinet.net; 4 Division of Urology, Department of Surgery, Taichung Veterans General Hospital, Taichung 40705, Taiwan; u99001099@gap.kmu.edu.tw (S.-J.C.); gu5121@vghtc.gov.tw (K.-Y.C.); 5 Department of Obstetrics and Gynecology, Department of Medical Research, Department of Urology, China Medical University Hospital, Taichung 40447, Taiwan; 6 Department of Psychology, College of Medical and Health Science, Asia University, Taichung 41354, Taiwan

**Keywords:** urinary bladder, animal model, incontinence, overactive bladder, interstitial cystitis

## Abstract

**Simple Summary:**

The treatment of urinary bladder dysfunction requires the knowledge of bladder function, which involves physiology, pathology, and even psychology. Several animal models are available to study a variety of bladder disorders. These models include animals from rodents, such as mice and rats, to nonhuman primates, such as rabbits, felines, canines, pigs, and mini pigs. This review adapted animal models to study bladder function according to facility, priority, and disease.

**Abstract:**

The urinary bladder (UB) serves as a storage and elimination organ for urine. UB dysfunction can cause multiple symptoms of failure to store urine or empty the bladder, e.g., incontinence, frequent urination, and urinary retention. Treatment of these symptoms requires knowledge on bladder function, which involves physiology, pathology, and even psychology. There is no ideal animal model for the study of UB function to understand and treat associated disorders, as the complexity in humans differs from that of other species. However, several animal models are available to study a variety of other bladder disorders. Such models include animals from rodents to nonhuman primates, such as mice, rats, rabbits, felines, canines, pigs, and mini pigs. For incontinence, vaginal distention might mimic birth trauma and can be measured based on leak point pressure. Using peripheral and central models, inflammation, bladder outlet obstruction, and genetic models facilitated the study of overactive bladder. However, the larger the animal model, the more difficult the study is, due to the associated animal ethics issues, laboratory facility, and budget. This review aims at facilitating adapted animal models to study bladder function according to facility, priority, and disease.

## 1. Introduction

The urinary bladder (UB) is an organ of the urinary system that stores and empties urine. Its storage volume is approximately 300–500 mL, implying all voided volume. UB dysfunction can result in voided volumes below or over this capacity and can cause disorders. Voiding symptoms include frequency of urination, urgency of urination, dysuria, pain, incontinence, and the inability to completely void urine, i.e., urinary retention. Voiding dysfunction often originates from complications related to neurons, muscles, infection, psychological stress, side effects of drugs, or pelvic disorders. The treatment of UB dysfunction relies on correcting the underlying factors.

Animal studies are pivotal to understand the underlying factors of UB. However, unlike most four-legged animals that sometimes urinate at random places, unless they are reared in a cage, humans usually void urine in an allocated place. This behavior implies that humans control bladder reflex by voluntarily contracting pelvic muscles and external urethral sphincter. This unwilling action might hinder the use of animal studies in research on human bladder function. Therefore, no ideal animal model exists that completely mimics the voiding behavior in humans and provides a multifactorial basis of bladder function. Nonetheless, animal models can be utilized to further our understanding of bladder dysfunction pathophysiology and to search for potential treatment options [1]. Using animal models allow us to analyze specific risk factors or elements that contribute to bladder function and improve further management. It is noteworthy that, in relation to animal ethics-related problems, alternative test methods to replace animal studies have been published in Taiwan since 2019 [2]. Alternatives to animal testing are widely accepted and implemented globally, including in the US, European Union, Canada, Japan, and Korea (website: http://nehrc.nhri.org.tw/taat/links.php accessed on 8 December 2021). Therefore, it is beneficial to look for alternative methods before conducting an animal study.

## 2. Bladder Function Study

Unlike the tonic contraction of other visceral organs, the UB controls both voluntary and involuntary micturition techniques in a switch-like manner. The UB function is controlled by the complex central nervous system (CNS), which involves learning and maturation. This complex pathway is involved in physiology and psychology, such as judgment, emotions, social situations, aging, and drugs. Moreover, signaling pathways transmit activating and deactivating signals to the UB via complex routes, such as sympathetic, parasympathetic, and somatic pathways. The CNS circuit also transmits to the urethra and striated urethral sphincter [3].

Urine storage in the bladder involves the relaxation or inhibition of the bladder detrusor muscles to maintain low pressure, which is achieved by the guarding reflex [4]. This reflex originates in the bladder synapse, with spinal tracts in the lumbosacral spinal cord of afferent nerves, and stimulates reflex firing in the sympathetic and somatic efferents to the smooth and striated elements of the bladder, thus maintaining continence [5,6].

Emptying urine from UB involves two reflexes, bladder–bladder and bladder–urethral. The complete voiding of urine is achieved by bladder contraction and urethral relaxation. Neurons involved in this function include direct, inverse, and on–off neurons [6]. When the UB has a full sensation, the bladder–bladder reflex is activated and is inhibited until voiding is socially appropriate. This excitatory mechanism is completed by bladder afferent nerves that are connected to the sacral spinal cord interneurons. This interneuron synapse involves preganglionic efferent parasympathetic nerves [7]. Once bladder contraction is initiated, the bladder–urethral reflex is activated by relaxing the smooth muscle of the proximal urethra to open the outlet. This involves bladder afferent nerves synapsing with urethral parasympathetic efferent nerves, thus completing this reflex [8].

Chronic somatic problems may originate from visceral organs, including the UB. Altered viscero-somatic reflexes have been studied by recording monosynaptic reflexes. An inhibitory response was observed by the distention of the UB in decerebrate animals. Studies by de Groat et al. on the bladder strip in rats found that the alpha1-adrenoceptor agonist enhanced the neurally evoked bladder contraction, which can be blocked by selective alpha1-adrenoceptor blockers [4,5]. In the rat and cat CNS, the alpha1-adreceptor mechanism can modulate sympathetic, parasympathetic, and somatic outflow to the UB. The inhibitory mechanisms of alpha 1 adrenoceptors in the rat spinal cord can reduce the voiding reflex, which is possibly also inhibited in the afferent part of the micturition pathway. More studies should focus on the viscero-somatic reflexes.

## 3. Study Diseases

Table 1 summarizes the UB disorders and study models, comparing their laboratory priorities, advantages, and disadvantages.

### 3.1. Overactive Bladder

Overactive bladder (OAB) is a storage dysfunction of the UB that is defined as urinary urgency, usually with increased urination frequency and nocturia, with or without urgency of urinary incontinence [9]. OAB influences quality of life and economics with bothersome symptoms. Currently, several animal models are available to study OAB [10]. These animal models can be categorized into two major types: induced and transgenic/genetic models.

The majority of OAB animal models are the induced type wherein OAB is experimentally induced to observe relevant pathological symptoms in healthy animals, including cats, rodents, dogs, rabbits, or nonhuman primates. Induced models can be separated into further categories as follows: peripheral versus central models, induced hypersensitivity/inflammation models, and bladder outlet obstruction (BOO) models [11].

#### 3.1.1. Peripheral versus Central Model

Peripheral models aim to present direct damage to the bladder nervous system, blood supply, or metabolic status [12]. Central models are induced surgically, or with the use of substances, to develop a series of injuries or damage in the brain, spinal cord, brain stem, or other parts of CNS [13,14]. The main disease status might not reflect OAB, but shares similar bladder symptoms.

Peripheral and central models are easy to create and can be applied in nervous system-related or metabolic system-related OAB, but cannot replicate idiopathic OAB. This includes neurological models, spinal cord injury/transection [15], and hyperlipidemic models [16]. Transection of T10 spinal cord to cause paraplegia and induce neurogenic detrusor overactivity in rats is an example of a central model. Rats fed with a high fat diet in a hyperlipidemic model to study bladder function causing OAB, erectile dysfunction, and prostate enlargement is an example of a peripheral model.

#### 3.1.2. Induced Hypersensitivity/Inflammation Models

These are most commonly used in OAB studies to induce hypersensitivity and inflammation through the instillation of an external substance, such as acetic acid, citric acid, capsaicin, cyclophosphamide (CYP), and alpha-bungarotoxin [17].

#### 3.1.3. BOO Model

BOO is commonly observed in aging males with benign prostatic enlargement, leading to storage symptoms that might persist even after the transurethral resection of prostate. BOO models surgically replicate outlet obstruction by partially or completely ligating the urethra or bladder neck [18] or post-orchiectomy subcutaneous injection of testosterone in rats, leading to physiological bladder wall changes in animals, including muscle cell hypertrophy or detrusor muscle denervation [11,12,18,19].

Partial BOO models are considered reliable to study lower urinary tract symptoms with etiological validity. Partial obstruction of urethra in animals causes several morphological and functional changes in bladder and nerve pathways, such as detrusor muscle hypertrophy, reduced bladder capacity, increased detrusor activity, collagen deposition in lamina propria, and changes in the neurotransmitter; all of these mimic the pathological changes in humans [20].

#### 3.1.4. Genetic Animal Model

Commonly seen in spontaneous hypertensive models [17], this model mimics human hypertension, showing bladder frequency and reduced bladder volume. Although the cause of bladder dysfunction is still unclear, it gives a glimpse of how central and peripheral systems affect micturition reflex control.

#### 3.1.5. Transgenic Model (Knock-In/Knockout)

Advanced genetic engineering is an advanced approach, and several transgenic mouse models have been developed to study the characteristics of lower urinary tract physiology and dysfunction. These are now common models, such as neuronal nitric oxide synthase, uroplakin, prostaglandin receptor, purinergic receptor, or estrogen-receptor knockout mice [21].

### 3.2. Incontinence

Urinary incontinence is defined as the involuntary loss of urine. Lin et al. used intravaginal balloon distension in female rats to mimic birth injury-related urinary incontinence. Vaginal distension damaged the muscular and neural structure in the pelvis, contributing to urinary incontinence [22]. Pudendal nerve injury is also responsible for birth trauma-related urinary incontinence; Kerns et al. used Dumont #5 forceps to bilaterally crush the pudendal nerve via the ischiorectal fossa, causing reversible dysfunction of the external urethral sphincter [23]. Rodriguez et al. created a durable urinary incontinence animal model through transabdominal urethrolysis. The urethra was skeletonized from the vaginal wall and pubic bone, resulting in decreased abdominal leak point pressure and retrograde urethral perfusion pressure, similar to intrinsic sphincter dysfunction [24].

**Figure 1:**
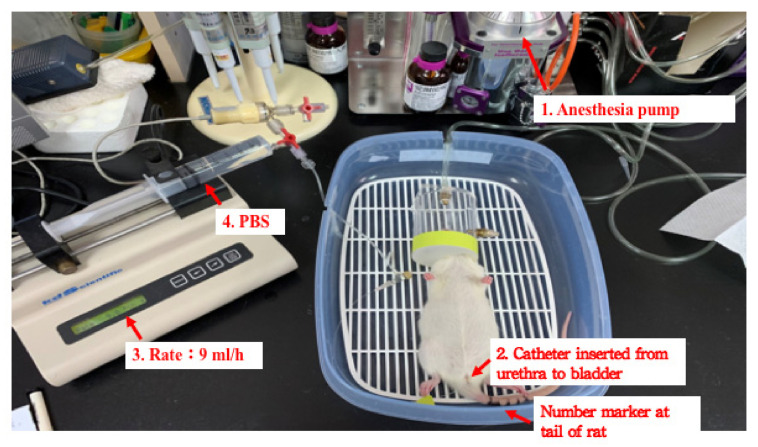
A representative image for the experimental set-up of leak point pressure measurement in a rat.

**Figure 2:**
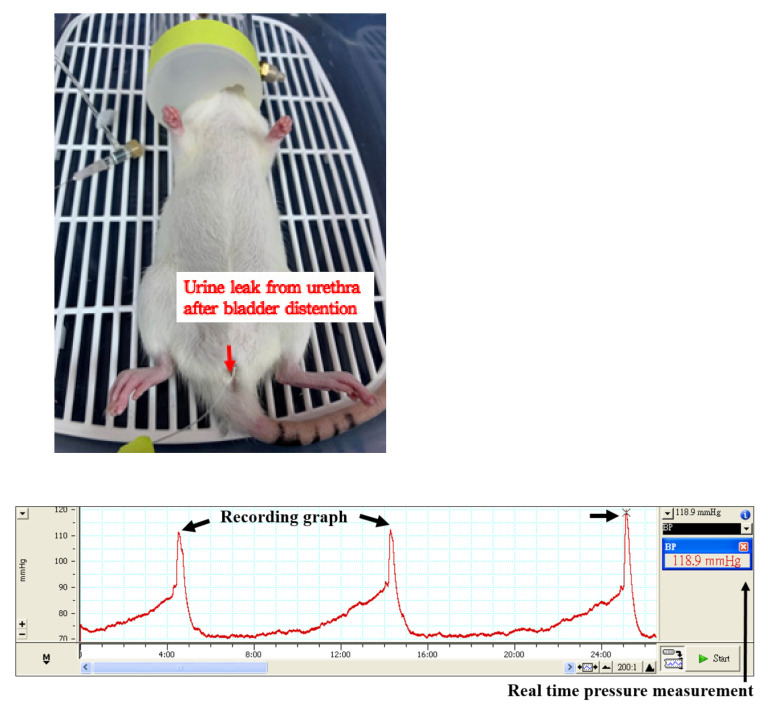
A representative experimental image for the real time recording of intravesical pressure during urinary bladder filling in a rat model.

The integral theory of stress urinary incontinence (SUI) was discovered by Petros and Ulmsten [25]. Based on this theory, Kefer et al. created an SUI animal model by the transection of pubo-urethral ligament via a suprapubic midline incision [26]. The leak point pressure significantly decreased after the pubo-urethral ligament transection. This model could be used to study urethral hypermobility-related SUI (Figure 1 and Figure 2 represent the experimental set-up of leak point pressure measurement in a rat, and the real time recording of intravesical pressure during UB filling in a rat model, respectively).

Obesity is also a risk factor for SUI. Zucker fatty (ZF) rat is a transgenic rat with a genetic mutation of the leptin receptor, resulting in hereditary obesity. Wang et al. demonstrated that the ZF rat exhibits higher voiding frequency and lower leak point pressure compared with Zucker lean rats [27].

Chen et al. used leak point pressure and maximal urethral closure pressure to evaluate SUI in ovariectomized mice [28]. Pathogenesis of SUI after menopause would open additional avenues for novel research and potential therapies.

### 3.3. Interstitial Cystitis/Painful Bladder Syndrome

The etiology of interstitial cystitis/painful bladder syndrome (IC/PBS) remains unclear and involves multiple factors. Therefore, animal models are created by noxious intravesical or systemic stimulation to mimic the complex human pathophysiology [29]. The following are the three animal models that mimicked the clinical features of IC/PBS patients: (1) bladder-centric models, (2) models with complex mechanisms, and (3) psychological and physical stressors/natural models [29].

In bladder-centric models, a toxic substance is either injected or administered to mimic an inflammatory condition. The commonly used animals in these studies are mice or rats. Rodent (mouse and rat) models of interstitial cystitis could be created by the administration of irritants into the UB, or the altered expression of the urothelium. The most commonly injected irritant agent in mice is CYP, which is used to study bladder neuronal pathway alterations in response to chronic inflammation [30,31,32]. In rats, low-dose systemic injection of CYP might cause severe bladder tissue inflammation and damage, which is atypical in human bladder pain syndrome [33,34]. Conversely, mice are more resistant to systemic CYP treatment and appear more suitable to study bladder pain syndrome [35]. Low-dose CYP injection in mice causes frequency and bladder overactivity without physiological impairment. The bladder tissue showed edema, hyperalgesia, lymphocyte infiltration, and urothelium hyperplasia [36]. Although hyperplasia is not a typical symptom of bladder pain syndrome, chronic systemic CYP injection in mouse models is still an important approach to study the underlying pathophysiology of bladder structures. However, rat bladder is more suitable for the study of bladder function, such as leak point pressure, as its capacity is more suitable than that of the mouse [37].

Pseudorabies virus tail injection activates the CNS, causing bladder pain syndrome; this is an alternative method [38]. The injection causes the activation of the CNS circuit of the specific neurons in rats that innervate the bladder, thus inducing localized immune response and bladder inflammation. This is a type of neurogenic cystitis. Intravesically delivering compressed air at a specific pressure to anesthetized mice, and recording the electrical activity of the superior oblique abdominal muscle, is another method to study IC/PBS [39]. This measurement is a reliable and reproducible of nociception as visceromotor response. It allows the investigation of the pain-involved coordination between primary sensory neurons, spinal cord secondary afferents, and the higher CNS.

A hypothetical explanation of IC/PBS is autoimmunity. Lin et al. proposed an animal model of experimental autoimmune cystitis [40]. Female mouse bladder was instilled mouse bladder homogenate to induce bladder immune responses. After a 4-month instillation, a histology analysis of harvested bladder tissue revealed thickened lamina propria, infiltration of lymphocytes, giant cells, and increased mast cells in the detrusor muscle. Frequent and decreased voided urine volume and voiding interval without change of peak voiding pressure could be observed in the mice. This model could be an acceptable murine model to study the human IC voiding pattern.

The third model of IC/PBS is a feline model of psychological stress. Water avoidance and phenylephrine are most recommended to induce psychological stress. The water-avoidance test involved female adult Wistar rats placed on a pedestal in a water-filled cage for 1 h per day for a week [41]. The mechanical stress pain threshold, urine level of norepinephrine cystometry, and bladder tissue histology of the rats were studied. The results revealed increased levels of norepinephrine, urinary frequency, and decreased pain threshold, all of which could be reversed by alpha-blockers. UB tissue strips were placed into a culture medium containing phenylephrine for 24 h to measure nerve growth factor levels. This model implies that alpha-adrenoceptors might play a role in IC/PBS.

### 3.4. BOO

The partial obstruction of bladder outlet can be surgically induced in both sexes of pigs, dogs, rabbits, guinea pigs, rats, and mice [42]. Buttyan et al. created an artificial partially obstructed rabbit model that mimics human benign prostate hyperplasia [43]. The partial obstruction was made using a 2-0 silk ligated loosely around the proximal urethra under urethral catheterization. The initial pathology was ischemia with the following three phases: hypertrophy, compensation, and decompensation of the UB. Growth factors, such as fibroblast growth factor, epidermal growth factor, and transforming growth factor beta, are involved in the hypertrophy phase. Due to persistent obstruction, the UB muscles degenerated and became dysfunctional in the decompensation phase.

Kitta et al. used female mice to induce partial BOO and observed the voiding behavior change in the circadian cycle in the long term (12 months) [44]. The partial obstruction method involved a metal rod (0.55 mm in diameter) placed beside the proximal urethra and tied using a 4-0 silk ligature around them. They used automated voided stain paper to precisely record the voiding pattern. The quantitative gene expression was also investigated by qRT-PCR at 12 months, which showed a 3.4-fold increase of the 5-HT2B receptor gene expression. This could be the cause of nocturia in humans as the circadian bladder function was disrupted involving gene overexpression and body weight gain.

### 3.5. Ketamine Cystitis

Ketamine is an *N*-methyl-d-aspartic acid receptor complex antagonist which is used as an anesthetic and analgesic. It has been illegally used for recreational purposes in Asian countries, including Taiwan, for over a decade [45]. Ketamine abuse resulted in 30% of users developing cystitis with pathologies such as interstitial cystitis/painful bladder syndrome, which includes features such as urothelial ulceration, inflammatory cell infiltration, and varying degrees of bladder wall fibrosis [46]. The clinical symptoms of ketamine cystitis (KC) include frequency, urgency, urinary incontinence, dysuria, and gross hematuria [45]. Several possible mechanisms could be involved in causing KC, including problems associated with microvascular system, neurotoxicity, autoimmunity, and metabolite norketamine direct toxicity, and the disruption of urothelial barrier [45,47,48].

Rajandram et al. used intraperitoneal ketamine injection on female mice for 12 weeks to induce KC [47] and observed a contracted and OAB. This successful experimental model of voiding dysfunction in mice did not show any disruption of urothelial barrier function. Therefore, the authors concluded that the barrier dysfunction might not be a major mechanism of KC. Shen et al. also used intraperitoneal ketamine injection in mice for 20 weeks and studied the cDNA microarray [49]. They also successfully induced KC in mice and found subepithelial congestion and lymphoplasmacytic aggregation and identified a number of genes associated with extracellular matrix accumulation, connective tissue fibrosis, and calcium signaling regulation. These findings suggested that the KC symptoms were related to the alteration of smooth muscle contraction and urothelial pathogenesis.

Lee et al. used a rat model to investigate the effect of hyaluronan on the bladder mucosal repair in KC [50]. They hypothesized that hyaluronan treatment can alter the bladder urothelial layer as well as the expression of hyaluronan-metabolizing enzymes and hyaluronan receptors. Their results were promising, and intravesical hyaluronan instillation has been widely used since then in the clinical setting to treat ketamine-induced cystitis [51,52,53]. Oral administration of the traditional Chinese medicine Ba-Wei-Die-Huang-Wan (BWDHW) was reportedly efficient in the treatment of cystitis in a ketamine-injected rat model [54]. BWDHW inhibits rats with KC induced the upregulation of neuroreceptors, inflammation, fibrogenesis, and bladder overactivity. Therefore, this medicine is a potential alternative, orally-administered agent to treat ketamine-induced cystitis. However, clinical trials to confirm the effects of BWDHW on the treatment of KC are warranted.

### 3.6. Limitations

Discussion regarding the use of animal models to experimentally study underactive bladder and mixed incontinence is lacking. Drug resistance bacterial infection of UB is also an important factor for the successful treatment of UB dysfunction. An experimental animal model to study this condition should be identified.

## 4. Study Animals

### 4.1. Guinea Pigs

The guinea pig (GP) is an ideal model to study the bladder smooth muscles [55,56]. GP has long been used to study human diseases, since it is small, inexpensive, and easy to house and care for. Under adequate anesthesia, GP could be used as a urodynamic study animal for simultaneous flow rate and pressure measurements [57], with similar results as in non-anesthetized animals. The bladder muscle strip in GPs is an excellent and suitable tissue to conduct electrophysiological studies on the detrusor muscle contraction via electrode recording [58]. A new model has been developed to study BOO-induced smooth muscle alteration by partial clamping of the GP urethra [59]. In this model, silver jump rings were applied around the urethra of immature GPs and with the growth of the animal, the urethra was gradually obstructed. Several weeks are required to allow the animals to reach maturity before examining UB obstruction.

The disadvantages of GP in the study of bladder function include small bladder muscle tissue, the purinergic component of GP neuromuscular transmission not being fully compatible with that of human (not responding well to atropine), and the cage required to house them being larger than those of mice.

### 4.2. Rabbits

Levin et al. introduced rabbits as a model to study bladder function [60]. The in vitro studies on bladder function in rabbits included cystoscopy, cystometry, micturition volume, and frequency of micturition measurements. Under pentobarbital anesthesia, the rabbit bladder acute urinary overdistention could be measured. The results revealed bladder muscle dysfunction of contraction, increased mucosa, permeability, and the proliferation response of the epithelium [61,62]. BOO also could be studied in rabbits though 1-0 silk ligation over a precatheterized urethra [63].

The bladder tissue strip or isolated whole urethra of rabbits can be used as an in vitro study system of bladder function [64,65]. The bladder strip responded to the stimulation of muscarinic cholinergic stimulation (bethanechol), beta adrenergic stimulation (isoproterenol), purinergic stimulation (ATP), and alpha-adrenergic agonist stimulation (methoxamine). Urethral segment could be used to measure the urethral pressure test alpha-blocker response via the stimulation of norepinephrine.

The rabbit model can be used for multipurpose studies of UB function both in vitro and in vivo. The disadvantages of using the rabbit model include relative larger animal size, the need for more care at the time of breeding, and frequent incidences of bladder stones, due to the inactivity of the animals in the cage, or animals holding urine due to inappropriate toilet area [66].

### 4.3. Cats

Cats show the ability to urinate in an allocated place, which is similar to humans. Cats reportedly exhibit multiple urinary tract disorders, including voiding symptoms [67]. Cats have a thicker bladder wall and are less compliant than rabbits [68], leading to them having relatively higher intravesical pressure to void urine. Therefore, cat animal models can be used to study SUI [1]. Urethral closing pressure can be measured in anesthetized female cats during sneezing-induced SUI [69,70]. Urodynamic studies in cats require anesthesia, which is different from awake humans. Therefore, the anesthesia agent used could be a confounder during urodynamic study. Xu et al. studied agent-specific differences in urodynamic studies on cats with anesthesia agents to elucidate this problem [71]. They found that propofol had a short induction time and high bladder pressure slope during the initial and final portion of cystometrogram. Dexmedetomidine had a short recovery time, high bladder pressure slope, and deep plane of anesthesia. This information allows researchers to make the appropriate choice of anesthesia during future studies.

In a study on spinal cord injury in the feline model, Yoo et al. found that selective stimulation of different pudendal nerves might have a potential micturition pathway to restore bladder function [72]. After adequate anesthesia, the spinal cords of four male cats were surgically transected at T10. Bladder contraction evoked by different branches of pudendal nerve was recorded via suprapubic catheterization of the bladder. Five male cats were used as controls to compare the contraction. The results revealed the dorsal genital branch of pudendal nerve that remained intact after spinal cord injury and bladder contraction responded to high frequency stimulation. This useful information might clinically potentially aid to restore bladder function in spinal cord injury patients.

Studying bladder function using cat models could have some disadvantages in terms of animal ethics. Anesthesia in cat increased risk of complications and death [73]. Therefore, greater care and attention should be taken during urodynamic study in cats. The cats in non-survival experiments should be euthanized with an overdose of pentobarbital.

### 4.4. Rats

Birth trauma is thought to cause SUI. Vaginal trauma can simulate birth trauma in a rat model. If active labor is prolonged for more than 30 min, the pressure on the vaginal wall during contractions can be as high as 240 cm of water column. High pressure maintained on the vaginal wall may cause microcirculation ischemia and excessive stretching of pelvic floor muscles, pubic-urethral ligaments, and pelvic nerve tissue; these combined events lead to SUI [74,75]. Given the scarcity of human urethral tissue available for analysis, the animal model of SUI induced by vaginal dilatation (VD) represents a reasonable alternative to studying the effects of childbirth on the urethra. Lin et al. first reported using an inflated balloon catheter in rat vagina for 4 h to simulate prolong labor [22]. After 4 weeks, the rate of stress incontinence was 20.8% (19/48 mouse). Huang et al. elongated the dilatation interval to 8 h and achieved to 72.5% (29/40 mouse) [76].

Leak point pressure and maximum urethral closure pressure are practical methods for assessing impaired urethral incontinence function after birth injury in animal models [77]. The urethral closure pressure (Pclose) is calculated by subtracting the urethral pressure (Pure) from the bladder pressure (Pves): Pclose = Pure − Pves [78]. Maximum urethral pressure and maximum urethral closure pressure can be obtained from the urethral pressure profile measurement (Figure 1).

The SUI animal studies have certain limitations. First, rats are quadrupeds with loose abdominal walls and different pelvic floor structures from that of humans; therefore, the results of animal studies on rats should be used with caution in human subjects. Second, it is clear that all aspects of human disease cannot be simulated by a single model. Multiple models may be required, each of which helps to reconstruct a reasonable picture of the pathophysiology and time course of SUI to determine reasonable treatment targets.

### 4.5. Mice

Mice are frequently used to study bladder function. Chen et al. applied and modified VD model to mimic birth trauma in mice. They dilated mice vagina with Hega’s dilator to induce SUI under anesthesia [79]. SUI decreased leak point pressure, overexpressed lysyl oxidase, decreased synthesis of extracellular matrix components, and increased proteolysis. Chen et al. further studied the estrogen-receptor knockout female mouse (ERα^−/−^) to investigate the role of estrogen play in SUI [80]. They found decreased leak point pressure and maximal urethral closure pressure in knockout mice. Proteomic study of mice’s urethra identified five down-regulated proteins and six up-regulated proteins involved in muscle development and contraction, proteolysis, cell adhesion and immune response. This information provided molecular mechanism of urethra responded to SUI. Synergistic VD and ovariectomy to study SUI in mice model was performed by Chen et al. [81]. Estrogen receptor and nitric oxide mediated signal pathways were found to be involved in the pathogenesis of SUI.

Sidler et al. reported a noninvasive model to assess murine bladder function [82]. They put mice in a modified metabolic cage and urine weight that allowed continuous recording with an alternative assessment of the voiding pattern. For avoiding urine stick to the collecting funnel, a paper-covered plate residing on a weight scale underneath the cage was replaced. This method has several advantages, such that it is low-cost, noninvasive, and suitable for long-term observation.

### 4.6. Canines

A combined cystourethrometry and urethral pressure profile can be performed in female dogs under adequate anesthesia for the simultaneous study of bladder storage function and urethral closure pressure [83]. This study examined continence and refractory incontinence female dogs for the evaluation of clinical effect of sling procedure.

A pilot study for the urodynamic investigation of female Beagle dogs was performed after surgical implantation of telemetric and electromyographic devices [84]. The device contains biopotential leads and thin wall catheters, which can be used to measure pressure, biopotentials, temperature, and activity. This method sends a record via radio wave able to reduce experimental animal stress due to noninvasive technique of prolonged monitoring and does not require animal handling. This method can be used for pharmacological studies. As urodynamic studies are conducted in the day and in the night, the effects of circadian rhythm also can be investigated.

### 4.7. Pigs

Whole UB of a female pig can be obtained from a local abattoir, which will enable the study of intravesical pressure in vitro [85]. After retrieval of the bladder, an intravesical drug can be administrated to measure the spontaneous movement of muscle with volume/pressure change. This model was also used in an ischemia model to study the bladder muscle autoregulation and the regulation of detrusor compliance [86,87]. Contractile dynamics of resting ex vivo pig bladder can be monitored and analyzed with video image recorder [88]. This spatiotemporal mapping of propagation patches of contractions model could contribute our understanding of the generation of resting tone of bladder. It is helpful to study clinical disorders such as OAB, painful bladder syndrome, and detrusor overactivity. Pigs also provide large amount bladder muscle strips for the pharmacological study of detrusor muscle contraction. We had the experience of using pig ureter to study the contraction of the ureter and we proved its advantages [89]. In addition, from a study of Mitsui et al. on the pig UB found that pig UB has muscularis mucosa that responded to the sympathetic stimulation of spontaneous phasic contraction [90]. Due to the lack of muscularis mucosa in rodents, this response is lacking in mouse and rat, which are less likely to completely mimic human bladder function. Time is also needed for the transportation of pig UB tissue from the local abattoir to the laboratory.

For a complex bladder function study, large animals such as pigs provide more informative data than small rodents, which give a valuable insight into the physiology of clinical conditions such as OAB and detrusor underactivity in vitro. Advantages also included zero breeding cost, easy availability, and large tissue sample. Pig models are likely to have disadvantages, such as large laboratory space, physiological solution preparation, and anesthesia.

### 4.8. Mini Pigs

Peterson et al. used bladder from mini pigs to study bladder contraction both in vitro and in vivo [91]. For the bladder wall substitution for the trauma, malignancy, and malfunction, Leonhäuser et al. used six adult female Göttingen mini pigs to compare two differently manufactured collagen scaffolds, OptiMaix 2D and 3D, as the material for bladder wall repair [92]. Unseeded scaffolds were initially implanted in mini pigs. After 6 weeks, the bladder wall was implanted with seeded scaffolds, tissue-cultured autologous urothelial cells, and detrusor muscle cells. The collagen scaffolds had good ingrowth capacity into the bladder wall and quickly linked to urothelial cells. This result was very promising for bladder wall augmentation. This relatively larger experimental animal has a large bladder volume and voiding frequency closely resembling that of humans. During the experiment, the functions of the mini pig bladder can be checked by contrast cystography, ultrasound, and cystometrography. However, this study used a normally functioning bladder instead of a diseased bladder. It was indicated that diseased bladders will be used in further studies. Breeding mini pigs in the laboratory requires a larger space and involves relatively higher costs.

## 5. Conclusions

Several disorders are caused by UB dysfunction, such as incontinence, OAB, interstitial cystitis, painful bladder syndrome, bladder outlet cystitis, and KC. Several animal models are available to study UB dysfunctions, including rodents, rabbit, dogs, pigs, and cats. However, no ideal model is available as yet that completely mimics the bladder function in humans. However, animal models can be selected from the models described above that best fit the research objective.

## Figures and Tables

**Table 1:** Comparison of the advantages and disadvantages of studying urinary bladder diseases in animal models. The animals are listed in order of laboratory priority.

Disease	Model	Animal	Advantage	Disadvantage
OAB	Peripheral/central damage model	Rat, mouse	Easy to produce OAB symptoms	Requires the surgical damage of CNS, disease status might not reflect OAB
	Hypersensitivity/inflammatory model	Rat, mouse	Most commonly used	
	Bladder outlet obstruction model	Dog, rat, mouse, rabbit, nonhuman primate	Mimics human pathophysiology	Requires surgical techniques
	Spontaneous hypertensive model	Rat	Mimics human hypertension	Unclear cause of bladder dysfunction
	Transgenic animal model	Mouse	Advanced approach	High cost of establishing transgenic animal models
Incontinence	Vaginal distension model	Rat, mouse	Low-cost and easy to handle	The pelvic anatomy of four-legged animals is not the same as that of humans and most models require surgical techniques
	Pudendal nerve injury model	Rat	Mimics human postoperative urinary dysfunction	
	Urethrolysis model	Rat	Mimics human urethral hypermobility-related stress urinary incontinence	
	Pubo-urethral ligament transection model	Rat, mouse	Mimics human urethral hypermobility-related stress urinary incontinence	
	Transgenic animal model	Rat	Mimics human obesity	High cost of establishing transgenic animal models
	Ovariectomy model	Mouse	Mimics menopausal model	Requires surgical techniques
IC/PBS	Bladder-centric model	Rat, mouse	Low-cost, easy to handle	Rat is atypical
	Pseudorabies virus tail injection model	Mouse	Reliable and reproducible model of nociception visceromotor response	Lack of muscularis mucosa in rodents
	Autoimmune cystitis model	Mouse	Mimics human IC voiding pattern	Requires long-term instillation
	Water-avoidance stress model	Cat, rat	Mimics psychological stress	
BOO	Ligated proximal urethral model	Pig, mouse, guinea pig, rat, dog, rabbit	Mimics human pathophysiology	Requires surgical techniques
	Post-orchiectomy injection of testosterone model	Rat		
Ketamine cystitis	IP model	Rat, mouse	Low-cost and easy to handle	Lack of muscularis mucosa in rodents

IC/PBS: interstitial cystitis/painful bladder syndrome; OAB: overactive bladder; BOO: bladder outlet obstruction; IP: intraperitoneal.
